# Impact of sugar beet pulp and wheat bran on serum biochemical profile, inflammatory responses and gut microbiota in sows during late gestation and lactation

**DOI:** 10.1186/s40104-021-00573-3

**Published:** 2021-04-20

**Authors:** Qinghui Shang, Sujie Liu, Hansuo Liu, Shad Mahfuz, Xiangshu Piao

**Affiliations:** State Key Laboratory of Animal Nutrition, College of Animal Science and Technology, China Agricultural University, No. 2 Yuanmingyuan West Road, Beijing, 100193 China

**Keywords:** Dietary fiber source, Gut microbiota, Inflammatory response, Serum biochemical profile, Sow

## Abstract

**Background:**

Sows are frequently subjected to various stresses during late gestation and lactation, which trigger inflammatory response and metabolic disorders. Dietary fiber can influence animal health by modulating gut microbiota and their by-products, with the effects depending upon the source of the dietary fiber. This study aimed to evaluate the impacts of different fiber sources on body condition, serum biochemical parameters, inflammatory responses and fecal microbiota in sows from late gestation to lactation.

**Methods:**

Forty-five multiparous sows (Yorkshire × Landrace; 3–6 parity) were assigned to 1 of 3 dietary treatments from d 85 of gestation to the end of lactation (d 21 post-farrowing): a control diet (CON, a corn-soybean meal diet), a sugar beet pulp diet (SBP, 20% SBP during gestation and 10% SBP during lactation), and a wheat bran diet (WB, 30% WB during gestation and 15% WB during lactation).

**Results:**

Compared with CON, supplementation of SBP decreased (*P* < 0.05) lactation BW loss, reduced (*P* < 0.05) serum concentration of total cholesterol, non-esterified fatty acids, interleukin-6 and tumor necrosis factor-α, and increased (*P* < 0.05) fecal water content on d 110 of gestation and d 21 of lactation, while supplementation of WB reduced (*P* < 0.05) serum concentration of total cholesterol on d 110 of gestation, increased (*P* < 0.05) fecal water content and decreased (*P* < 0.05) serum interleukin-6 concentration on d 110 of gestation and d 21 of lactation. In addition, sows fed SBP had lower (*P* < 0.01) abundance of *Clostridium_sensu_stricto_1* and *Terrisporobacter* than those fed CON, but had greater (*P* < 0.05) abundance of *Christensenellaceae_R-7_group* and *Ruminococcaceae_UCG-002* than those fed the other two diets on d 110 of gestation. On d 21 of lactation, supplementation of SBP decreased (*P* < 0.05) the abundance of Firmicutes and *Lactobacillus*, but enriched (*P* < 0.05) the abundance of *Christensenellaceae_R-7_group*, *Prevotellaceae_NK3B31_group*, *Ruminococcaceae_UCG-002*, *Prevotellaceae_UCG_001* and *unclassified_f__Lachnospiraceae* compared with WB. Compared with CON, sows fed SBP had greater (*P* < 0.05) fecal concentrations of acetate, butyrate and total SCFAs during gestation and lactation, while sows fed WB only had greater (*P* < 0.05) fecal concentration of butyrate during lactation.

**Conclusions:**

Supplementation of dietary fiber during late gestation and lactation could improve sow metabolism and gut health, and SBP was more effective than WB.

## Background

Pregnant sows are frequently subjected to psychological and physiological stresses (e.g. rapid fetal development and feed restriction), which can result in oxidative stress and metabolic disorders, and consequently an imbalance inflammatory response [1, 2]. Furthermore, labor-induced injury of the birth canal and uterus can exacerbate oxidative stress and inflammatory responses at parturition [3]. Moreover, even during lactation, the drastic catabolism and anabolism due to milk synthesis also can further contribute to metabolic disorders and inflammatory responses in sows [4, 5]. Long-term exposure to inflammation may, in turn, induce poor health status or even diseases [6]. Therefore, it is necessary to develop strategies to alleviate inflammatory responses and metabolic disorders in sows, especially during late gestation and lactation.

Recently, gut microbiota has been considered as an important factor of health due to its various effects on host [7]. A well-balanced microbiota plays a critical role in maintaining metabolic homeostasis and simulating immune system development [8]. In contrast, an imbalanced microbiota usually leads to poor performance, gut inflammation and metabolic disorders [9]. Generally, whether gut microbiota is beneficial to host health mainly depend on its metabolites derived from fermentation of indigestible substances in diets [10]. As a consequence, manipulation of gut microbiota and its metabolites by dietary modulation may be a potentially effective approach to improve sow health.

Dietary fiber (DF) is a mixture of carbohydrates that are indigestible by host enzymes but subjected to microbial fermentation, generating short chain fatty acids (SCFAs), principally acetate, propionate, and butyrate [11]. These SCFAs derived from DF fermentation, especially butyrate, have been demonstrated to have multiple health benefits, including increase insulin sensitivity, regulate immune system and reduce inflammation [10]. Previous studies with sows primarily focused on the beneficial effects of DF from a welfare perspective [12, 13]. In addition, some other studies showed that high fiber diets could improve the reproductive performance of sows [14]. However, the study [14] has not studied the influence of dietary fiber on gut health and the response to high-fiber diets may be variable due to different physiochemical properties of dietary fiber [15]. Sugar beet pulp (SBP) is a pectin-rich soluble fiber source, which is highly fermentable and has been shown to prevent post-weaning diarrhea by modulating gut microbiota composition in weaned pigs in some studies [16, 17]. Wheat bran (WB) is a source of insoluble fiber, rich in arabinoxylan and cellulose, which also has been shown to alleviate gut inflammation and enhance gut barrier function by improving gut microbiota in mice and weaned pigs [18, 19]. Till now, research with WB and SBP as a source of DF on sow’s health status during late gestation and lactation are limited. We hypothesized that supplementation of WB or SBP during late gestation and lactation would have different impacts on sow’s health via differently influencing microbiota. Therefore, the present study aimed to investigate the effects of supplementing the two sources of DF to sow diets during late gestation and lactation on body condition, serum biochemical parameters, immune responses, fecal microbiota and SCFAs.

## Materials and methods

### Animals, diets and management

Animal management and experimental procedures were approved by the Institutional Animal Care and Use Committee of China Agricultural University (Beijing, China). Forty-five healthy multiparous sows (Yorkshire × Landrace; 3–6 parity) were assigned to 1 of 3 dietary treatments balancing for parity, body weight (BW) and backfat thickness from d 85 of gestation to the end of lactation (d 21 post-farrowing). Dietary treatments included a control diet (CON, a corn-soybean meal basal diet), a sugar beet pulp diet (SBP, 20% sugar beet pulp during gestation and 10% sugar beet pulp during lactation), and a wheat bran diet (WB, 30% wheat bran during gestation and 15% wheat bran during lactation). The experimental two fiber diets had almost similar content of total dietary fiber. All diets were formulated to meet or exceed the nutrients requirements for sows as recommended by NRC (2012) (Table 1) [20]. The details nutrient composition of wheat bran (WB) and sugar beet pulp (SBP) are presented in the footnotes of Table 1.

**Table 1:** Ingredients and nutrient composition of experimental diets (%, as-fed basis)

Items	Gestation			Lactation		
	CON	SBP	WB	CON	SBP	WB
Corn	73.65	54.55	50.80	69.74	59.05	54.98
Soybean meal	22.00	21.50	15.00	26.00	26.00	23.00
Wheat bran^a^	–	–	30.00	–	–	15.00
Sugar beet pulp^b^	–	20.00	–	–	10.00	–
Soybean oil	0.85	0.85	0.85	0.65	1.53	3.50
Dicalcium phosphate	1.28	1.35	0.57	1.45	1.50	1.10
Limestone	1.07	0.60	1.48	0.78	0.54	0.97
Salt	0.40	0.40	0.40	0.40	0.40	0.40
*L*-Lysine HCl	–	–	0.15	–	–	0.06
Valine	–	–	–	0.23	0.23	0.24
Premix^c^	0.50	0.50	0.50	0.50	0.50	0.50
Chromium oxide	0.25	0.25	0.25	0.25	0.25	0.25
Calculated composition						
Digestible energy, kal/kg	3353	3259	3037	3388	3388	3388
Available phosphorus	0.31	0.31	0.31	0.34	0.34	0.34
SID lysine	0.69	0.69	0.70	0.78	0.79	0.78
SID methionine	0.22	0.19	0.20	0.23	0.22	0.22
SID threonine	0.49	0.46	0.45	0.53	0.52	0.51
SID tryptophan	0.14	0.14	0.13	0.16	0.16	0.16
SID valine	1.05	1.04	1.02	0.85	0.85	0.85
Analyzed composition						
Crude protein	15.29	15.41	15.48	17.21	17.25	17.21
Calcium	0.74	0.76	0.75	0.68	0.70	0.68
Total phosphorus	0.56	0.55	0.58	0.60	0.59	0.62
Total dietary fiber	11.37	21.60	21.81	11.82	16.83	16.88
Soluble dietary fiber	1.39	4.06	1.86	1.43	2.72	1.69
Insoluble dietary fiber	9.98	17.54	19.95	10.39	14.11	15.18

^a^Nutrient composition of wheat bran: dry matter, 89.37%; organic matter, 83.55%; crude protein, 17.12%; gross energy, 17.01 MJ/kg; neutral detergent fiber, 37.36%; acid detergent fiber, 11.55%; total dietary fiber, 44.57%; soluble dietary fiber, 3.89%; insoluble dietary fiber, 40.68%

^b^Nutrient composition of sugar beet pulp: dry matter, 91.42%; organic matter, 84.63%; crude protein, 10.29%; gross energy, 15.62 MJ/kg; neutral detergent fiber, 38.25%; acid detergent fiber, 23.48%; total dietary fiber, 61.69%; soluble dietary fiber, 17.12%; insoluble dietary fiber, 44.57%

^c^Premix provided per kilogram of diet: Gestation: vitamin A, 11,000 IU; vitamin D_3_, 1500 IU; vitamin E, 15 IU; vitamin K_3_, 1.6 mg; vitamin B_1_, 1.5 mg; vitamin B_2_, 3.0 mg; vitamin B_6_, 1.5 mg; vitamin B_12_, 0.015 mg; niacin, 22.5 mg; D-pantothenic acid, 15 mg; folic acid, 2.5 mg; biotic, 0.2 mg; Fe, 85 mg; Cu, 7.5 mg; Zn, 75 mg; Mn, 35 mg; I, 0.5 mg; Se, 0.3 mg; Lactation: vitamin A, 6500 IU; vitamin D_3_, 1550 IU; vitamin E, 15.5 IU; vitamin K_3_, 1.6 mg; vitamin B_1_, 1.6 mg; vitamin B_2_, 3.1 mg; vitamin B_6_, 1.5 mg; vitamin B_12_, 0.015 mg; niacin, 23 mg; D-pantothenic acid, 15.5 mg; folic acid, 2.5 mg; biotin, 0.2 mg; Fe, 85 mg; Cu, 10 mg; Zn, 100 mg; Mn, 50 mg; I, 0.5 mg; Se, 0.3 mg

*CON* control diet; *SBP* sugar beet pulp diet; *WB* wheat bran diet; *SID* standardized ileal digestible

During gestation, all sows were housed in individual gestation stalls (2.1 m × 0.6 m), and fed twice a day at 08:00 and 16:00 h. On d 106 of gestation, sows were transferred to the farrowing rooms where they were housed in individual farrowing crates (2.1 m × 1.5 m) on d 107 of gestation. During gestation, sows were, and water was freely available. To achieve the same digestible energy intake per day, sows were fed 3.00 kg/d of CON, 3.09 kg/d of SBP, and 3.31 kg/d of WB, respectively. On the day of farrowing, sows were fed 0.5 kg of lactation diets, and then feed allowance was gradually increased by 1.0 kg/d until ad libitum feeding. All sows also had free access to water during lactation. Within 24 h after farrowing, the litter size was standardized to approximately 11 piglets by cross-fostering within treatment.

### Sample collection

Individual body weight and backfat thickness at the last rib were recorded for sows on d 85 and 110 of gestation, within 24 h after farrowing, and at weaning. On d 110 of gestation and d 21 of lactation, blood samples were collected from 6 sows each treatment via the ear vein before feeding. Serum was isolated by centrifugation at 3000×*g* and 4 °C for 10 min, and frozen at − 80 °C until analysis. On d 110 of gestation and d 21 of lactation, 5 sows per treatment with same parity of 3 were selected and then fresh feces were collected directly by massaging the rectum, and immediately stored at − 80 °C until analysis.

### Fecal water content

Approximately 200 g of fecal samples were oven-dried at 103 °C for 72 h. The sample weight before and after oven-dried was recorded to calculate fecal water content.

### Serum parameters

Serum samples were thawed at 4 °C and mixed thoroughly before analysis. Serum biochemical parameters including urea nitrogen (UN), total cholesterol (TC), triglyceride (TG), non-esterified fatty acids (NEFA) and glucose (GLU) were measured by the commercial kits (Beijing Sino-uk Institute of Biological Technology, Beijing, China) using an automatic biochemical analyzer (Hitachi 7160, Hitachi High-Technologies Corporation, Tokyo, Japan).

Serum concentrations of immunoglobulins including IgA, IgG and IgM and inflammatory cytokines including interleukin-6 (IL-6), interleukin-10 (IL-10) and tumor necrosis factor-α (TNF-α) were measured by enzyme-linked immunosorbent assay kits according to the manufacturer’s instructions (Beijing Sino-uk Institute of Biological Technology, Beijing, China).

### DNA extraction and 16S RNA sequencing

Bacterial DNA was extracted from fecal samples using a Stool DNA Kit (Omega Bio-tek, Norcross, GA, USA) following the manufacturer’s recommendations. The DNA concentration was quantified by NanoDrop 2000 UV-vis spectrophotometer (Thermo Scientific, Wilmington, United States), and the integrity of DNA was checked by 1% agarose gel electrophoresis. The V3-V4 hypervariable region of 16S rRNA gene was amplified with primers F338 (5′-ACTCCTACGGGAGGCAGCAG-3′) and R806 (5′-GGACTACHVGGGTWTCTAAT-3′). Then Amplicons were extracted from 2% agarose gels, and purified using the AxyPrep DNA Gel Extraction Kit (Axygen Biosciences, Union City, CA, USA) and quantified with a QuantiFluor TM-ST fluorometer (Promega, USA). Purified amplicons were pooled in equimolar concentrations and paired-end sequenced (2 × 300) on an Illumina MiSeq platform according to the standard protocols. Demultiplexing and quality-filtering of raw sequences were performed by QIIME (version 1.17) with the following criteria: 1) The 300 bp reads were truncated at any site receiving an average quality score of < 20 over a 50-bp sliding window, and the truncated reads shorter than 50 bp were discarded, reads containing ambiguous characters were also discarded; 2) Only overlapping sequences longer than 10 bp were assembled according to their overlapped sequence. The maximum mismatch ratio of overlap region is 0.2. Reads that could not be assembled were discarded; 3) Samples were distinguished according to the barcode and primers, and the sequence direction was adjusted, exact barcode matching, 2 nucleotide mismatch in primer matching. Then the rest high-quality sequences were clustered into operational taxonomic units (OTU) at 97% similarity by UPARSE and chimeric sequences were identified and removed by UCHIME. Each 16S rRNA gene sequence was taxonomically allocated on the basis of the silva (SSU128) 16S rRNA database by RDP Classifier (http://rdp.cme.msu.edu/) with a confidence threshold of 70%.

### Fecal short chain fatty acids

Fecal concentrations of SCFAs were analyzed as previously described by Shang et al. [2]. Briefly, approximately 0.5 g of fecal samples were diluted in 8 mL ultrapure water, homogenized by ultrasonic oscillation, and centrifuged at 12,000×*g* for 10 min. Then the supernatant was diluted 50 times, filtered through a 0.22-mm filter, and 1.5 mL of supernatant was analyzed by a high-performance ion chromatography of ICS-3000 (Dionex, United States). The concentrations of SCFAs were expressed as mg/g of feces.

### Statistical analysis

Date were analyzed using SAS 9.2 (SAS Inst. Inc., Cary, NC, USA) with individual sow as an experimental unit. The relative abundance of gut microbial communities was analyzed by Kruskal-Wallis test. Other date were analyzed using GLM procedures followed by Tukey’s tests. Significant difference was declared at *P* < 0.05, and tendency was declared at 0.05 ≤ *P* < 0.10.

## Results

### Sow body condition

Effects of fiber sources on sow body condition are presented in Table 2. No significant differences were observed in sow BW (d 85 and 110 of gestation, d 1 and 21 of lactation) and backfat thickness (d 85 of gestation, d 1 and 21 of lactation). However, the lactation BW loss was decreased (*P* < 0.05) in sows fed SBP diets when compared with those fed CON diets, but similar with those fed WB diets.

**Table 2:** Effects of fiber sources on sow body condition

Item	CON	SBP	WB	SEM	*P*-value
Sow BW, kg					
d 85 gestation	232.5	235.7	228.7	4.35	0.53
d 110 gestation	253.6	258.0	251.7	4.03	0.52
Gestation gain	21.1	22.4	23.0	1.89	0.78
d 1 of lactation	228.2	233.6	228.1	4.15	0.56
Weaning	216.0	223.6	216.9	4.17	0.38
Lactation loss	12.2^a^	10.0^b^	11.1^ab^	0.56	0.03
Backfat thickness, mm					
d 85 of gestation	13.53	13.80	13.47	0.60	0.92
d 1 of lactation	14.67	15.00	14.53	0.65	0.87
Gestation gain	1.13	1.20	1.07	0.24	0.92
Weaning	12.07	12.93	12.27	0.68	0.65
Lactation loss	2.60	2.07	2.27	0.23	0.27

^a-b^Mean values within a row with different letters differ at *P* < 0.05

*CON* control diet; *SBP* sugar beet pulp diet; *WB* wheat bran diet

### Fecal water content

**Fig. 1:**
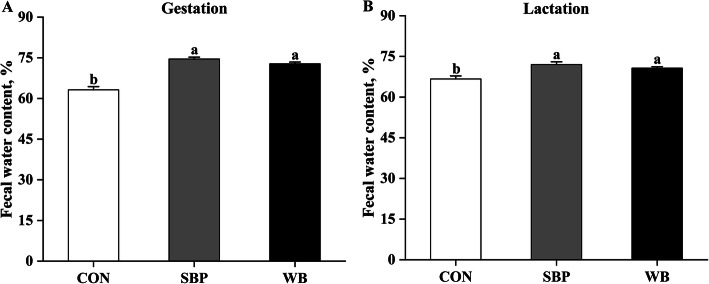
Effects of fiber sources on fecal water content in sows. (A-B) Fecal water content on d 110 of gestation and d 21 of lactation. Date were presented as mean ± SEM, *n* = 6. Different letters mean significant differences (*P* < 0.05). CON, control diet; SBP, sugar beet pulp diet; WB, wheat bran diet

Effects of fiber sources on fecal water content in sows are presented in Fig. 1. Both SBP and WB supplementation increased (*P* < 0.05) fecal water content in sows on d 110 of gestation (Fig. 1a). The same response was also observed on d 21 of lactation (Fig. 1b).

### Serum biochemical parameters

**Fig. 2:**
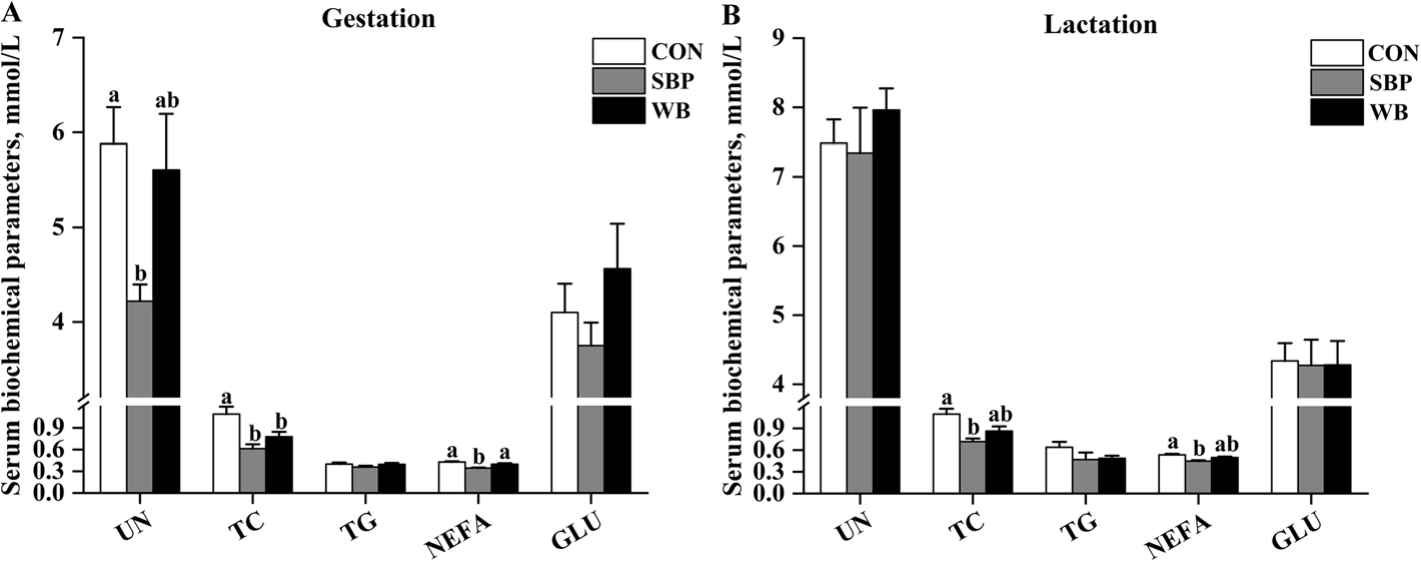
Effects of fiber sources on serum biochemical parameters in sows. **a**-**b** Serum biochemical parameters on d 110 of gestation and d 21 of lactation. Date were presented as mean ± SEM, *n* = 6. Different letters mean significant differences (*P* < 0.05). UN, urea nitrogen; TC, total cholesterol; TG, total triglycerides; NEFA, non-esterified fatty acids; GLU, glucose; CON, control diet; SBP, sugar beet pulp diet; WB, wheat bran diet

Effects of fiber sources on serum biochemical parameters in sows are presented in Fig. 2. On d 110 of gestation, a significant decrease (*P* < 0.05) in serum concentration of UN was observed in sows fed SBP when compared with those fed CON. Both sources of fiber supplementation significantly reduced (*P* < 0.05) serum TC concentration. Moreover, sows fed SBP showed a lower (*P* < 0.05) serum concentration of NEFA than those fed the other two diets. No significant differences were detected in serum concentrations of TG and GLU among treatments. On d 21 of lactation, serum concentration of UN was no longer changed by fiber supplementation. However, serum concentrations of TC and NEFA were still decreased (*P* < 0.05) by SBP supplementation when compared with CON. There was still no change in serum concentrations of TG and GLU among treatments.

### Serum immunoglobulins

**Fig. 3:**
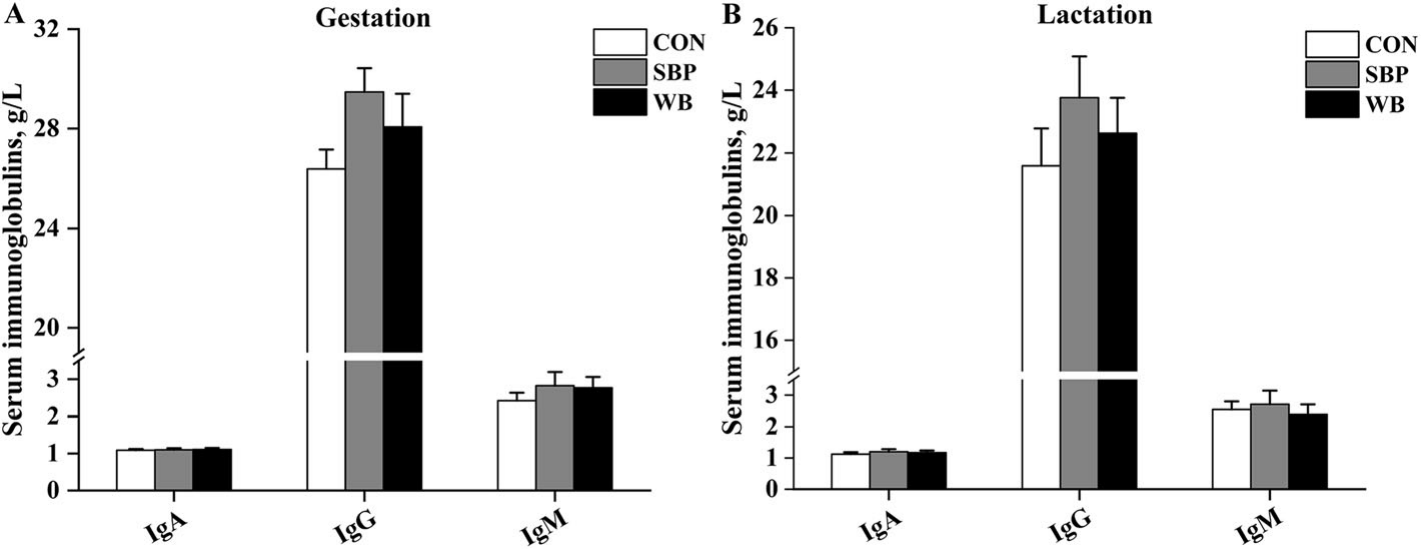
Effects of fiber sources on serum immunoglobulins in sows. **a-b** Serum immunoglobulins on d 110 of gestation and d 21 of lactation. Date were presented as mean ± SEM, *n* = 6. Different letters mean significant differences (*P* < 0.05). IgA, immunoglobulin A; IgG, immunoglobulin G; IgM, immunoglobulin M; CON, control diet; SBP, sugar beet pulp diet; WB, wheat bran diet

Effects of fiber sources on serum immunoglobulins in sows are presented in Fig. 3. Dietary treatments did not alter serum concentrations of IgA, IgG and IgM on d 110 of gestation or on d 21 of lactation (Fig. 3a and b).

### Serum inflammatory cytokines

**Fig. 4:**
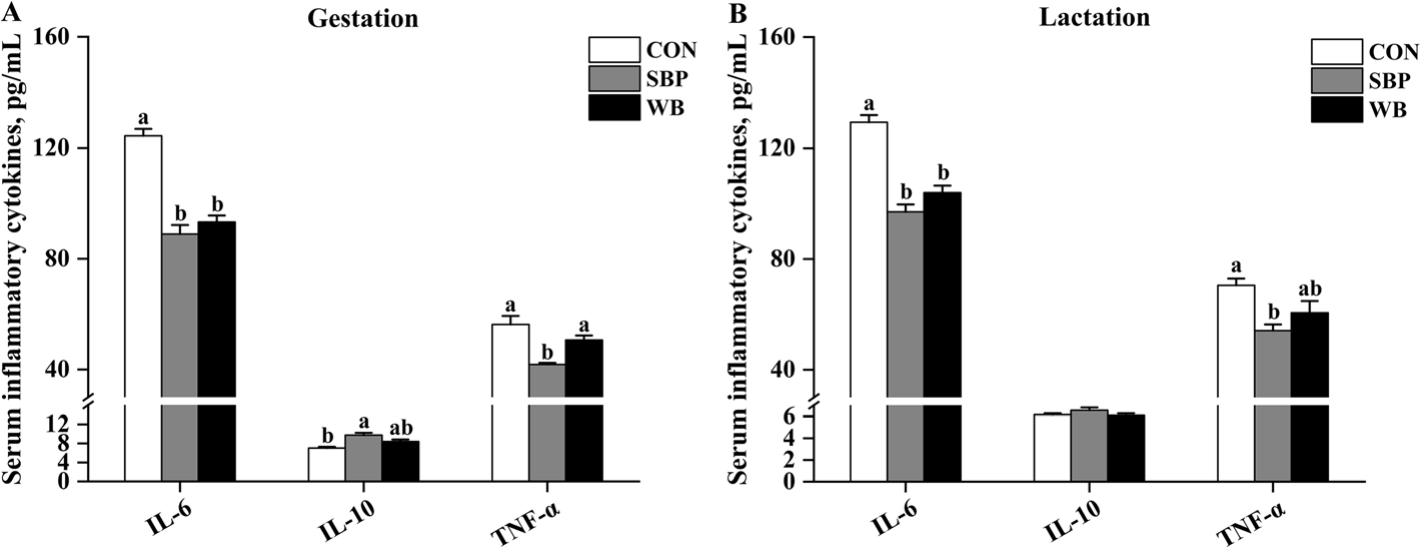
Effects of fiber sources on serum inflammatory cytokines in sows. **a-b** Serum inflammatory cytokines on d 110 of gestation and d 21 of lactation. Date were presented as mean ± SEM, *n* = 6. Different letters mean significant differences (*P* < 0.05). IL-6, interleukin-6; IL-10, interleukin-10; TNF-α, tumor necrosis factor-α; CON, control diet; SBP, sugar beet pulp diet; WB, wheat bran diet

Effects of fiber sources on serum inflammatory cytokines in sows are presented in Fig. 4. On d 110 of gestation, supplementation of both sources of fiber decreased (*P* < 0.05) serum concentration of IL-6 when compared with CON (Fig. 4a). Sows fed SBP showed greater (*P* < 0.05) serum concentration of IL-10 than those fed CON, but not different from those fed WB. In addition, the SBP supplementation decreased (*P* < 0.05) serum TNF-α concentration compared with the other two treatments. On d 21 of lactation, the decreased serum IL-6 concentration was also observed in sows fed SBP and WB than those fed CON (*P* < 0.05) (Fig. 4b). But dietary treatments did not influence serum IL-10 concentration. Serum TNF-α concentration was lower (*P* < 0.05) in sows fed SBP than those fed CON, but was similar to those fed WB.

### Fecal microbiota

**Fig. 5:**
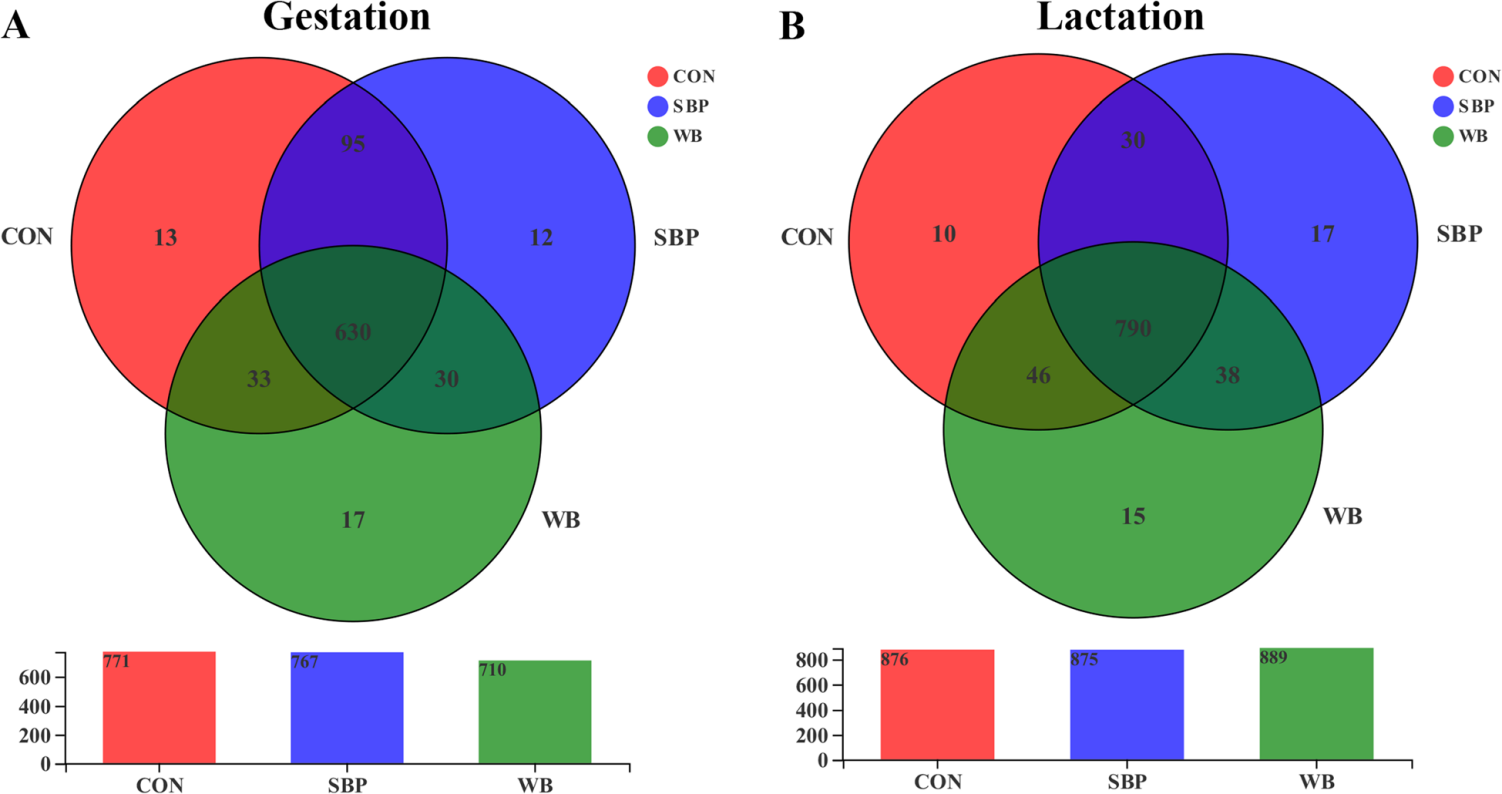
Venn diagram of the operational taxonomic units (OTUs) in sow feces on d 110 of gestation (**a**) and d 21 of lactation (**b**). *n* = 5. CON, control diet; SBP, sugar beet pulp diet; WB, wheat bran diet

**Fig. 6:**
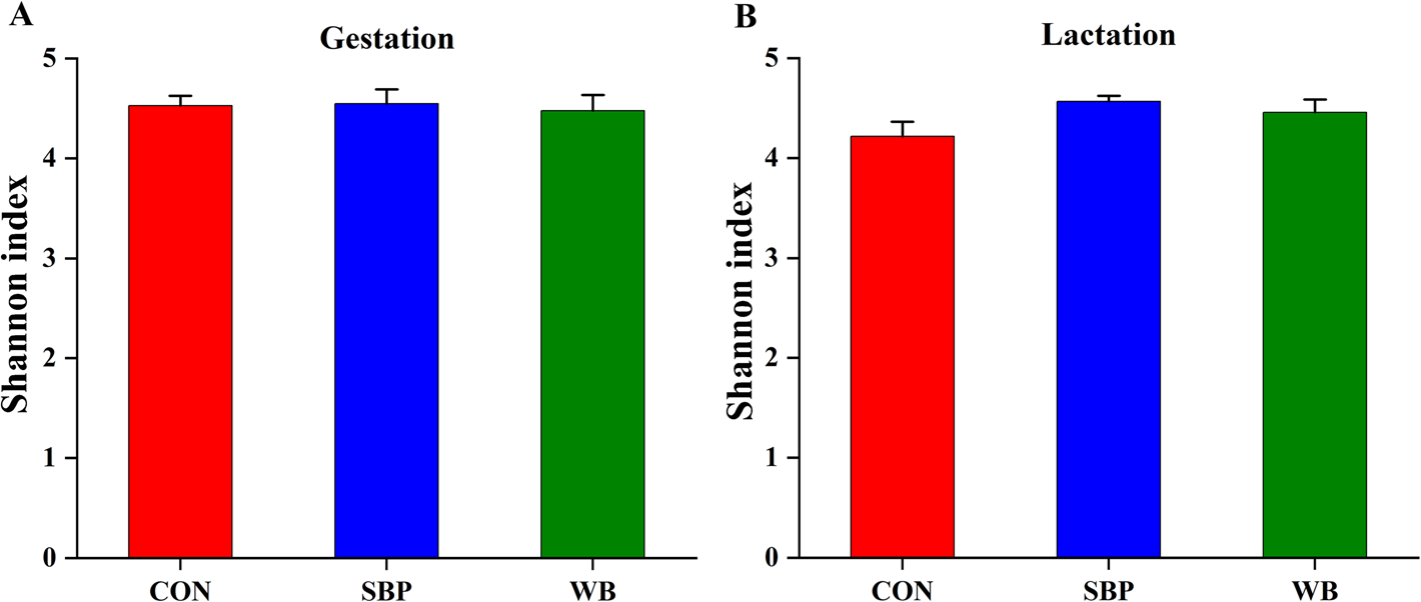
Alpha diversity of fecal microbial community determined by Shannon index on d 110 of gestation (**a**) and d 21 of lactation (**b**). *n* = 5. CON, control diet; SBP, sugar beet pulp diet; WB, wheat bran diet

**Fig. 7:**
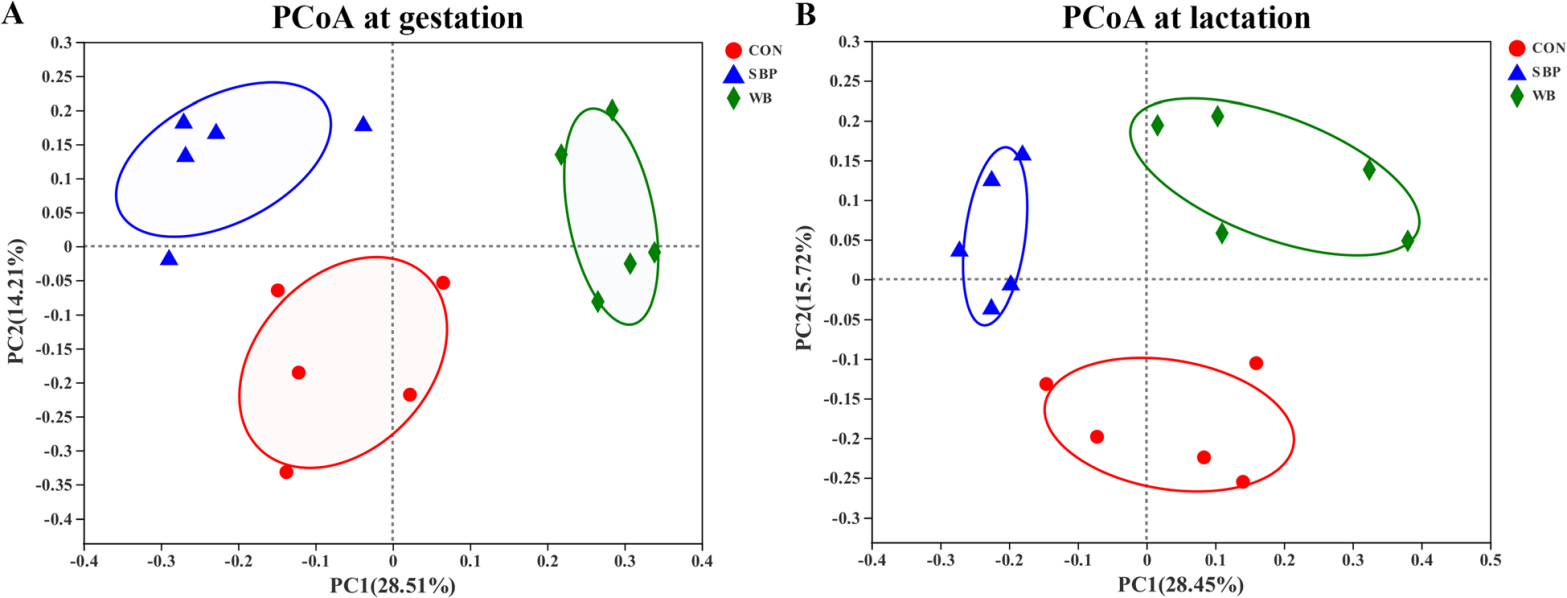
Principal coordinate analysis (PCoA) at the operational taxonomic unit (OTU) level based on Bray-Curtis dissimilarity on d 110 of gestation (**a**) and d 21 of lactation (**b**). *n* = 5. CON, control diet; SBP, sugar beet pulp diet; WB, wheat bran diet

To understand the effects of fiber sources on gut microbiota, 16S rRNA gene sequencing of fecal samples were performed. After quality control, a total of 538,051 and 825,315 high-quality sequences were generated from 15 fecal samples on d 110 of gestation and d 21 of lactation, respectively. The average numbers of high-quality sequences generated per sample were 35,870 and 55,021 on d 110 of gestation and d 21 of lactation, respectively. The Venn diagram showed that there were 771, 767, and 710 OTUs obtained from sows fed CON, SBP and WB on d 110 of gestation, of which 630 OTUs were shared and 42 OTUs were unique (Fig. 5a). There were 876, 875, and 889 OTUs obtained from sows fed CON, SBP and WB on d 21 of lactation, of which 790 OTUs were shared and 42 OTUs were unique (Fig. 5b). The bacterial alpha-diversity (Shannon index) was not significant different among treatments within each period (Fig. 6a and b). Principal coordinate analysis (PCoA) based on Bray–Curtis dissimilarity revealed that there was a clear separation of the microbial community among three treatments on d 110 of gestation and d 21 of lactation, indicating a shift in gut microbial communities (Fig. 7a and b).

**Fig. 8:**
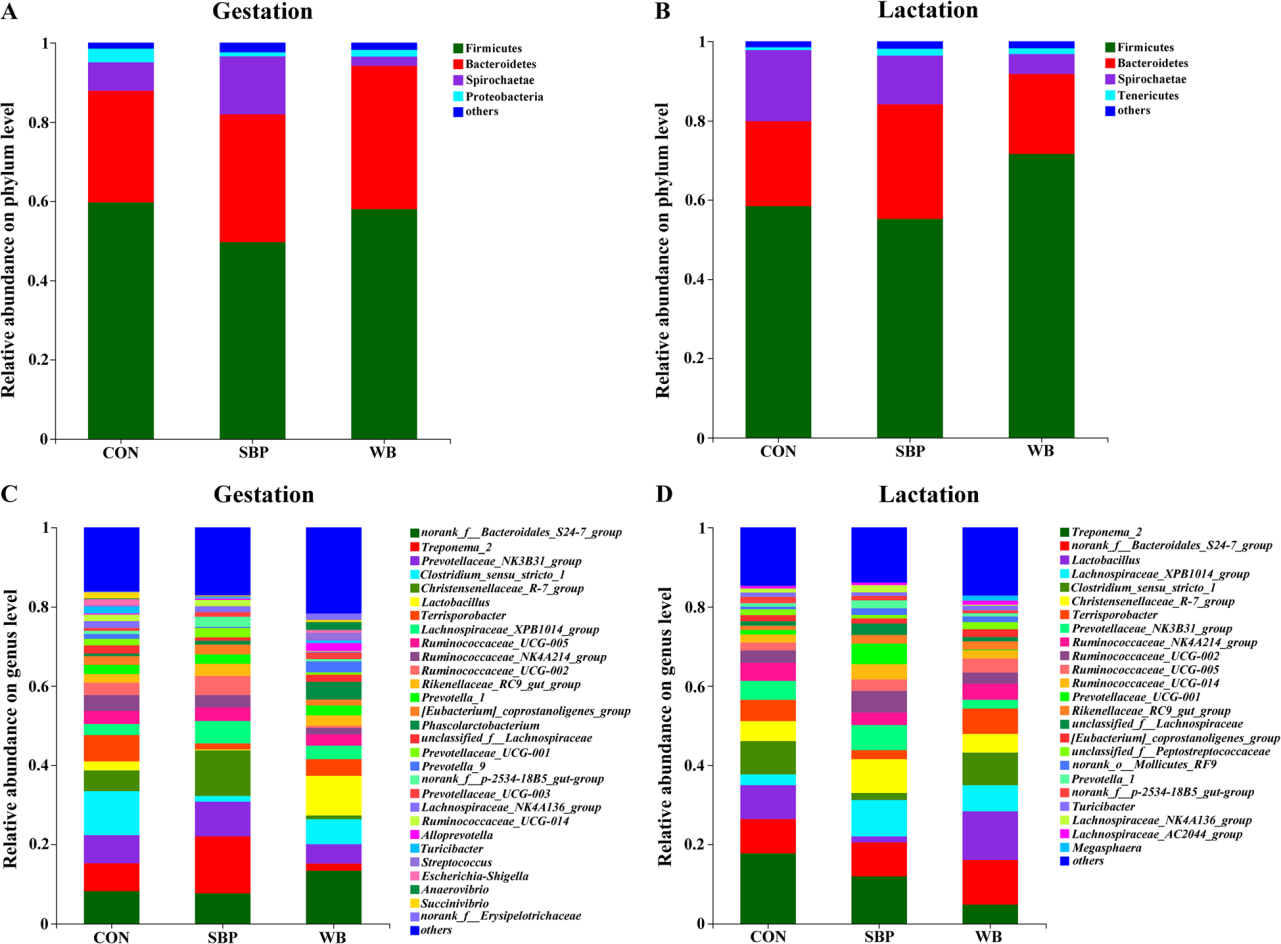
Effects of fiber sources on fecal microbiota composition in sows. **a-b** Microbial community bar plot at the phylum level on d 110 of gestation and d 21 of lactation. **c-d** Microbial community bar plot at the genus level on d 110 of gestation and d 21 of lactation. *n* = 5. CON, control diet; SBP, sugar beet pulp diet; WB, wheat bran diet

Subsequently, the effects of fiber sources on gut microbial composition in sows were investigated. At the phylum level, the dominant phyla during both periods were Firmicutes, Bacteroidetes, and Spirochaetes, accounting for more than 95% (Fig. 8a and b). On d 110 of gestation, the top three genera in CON were *Clostridium_sensu_stricto_1*, *norank_f__Bacteroidales_S24-7_group*, and *Prevotellaceae_NK3B31_group*; those in SBP were *Treponema_2*, *Christensenellaceae_R-7_group* and *Prevotellaceae_NK3B31_group*; and those in WB were *norank_f__Bacteroidales_S24-7_group*, *Lactobacillus* and *Clostridium_sensu_stricto_1* (Fig. 8c). On d 21 of lactation, the top three genera in CON were *Treponema_2*, *norank_f__Bacteroidales_S24-7_group* and *Lactobacillus*; those in SBP were *Treponema_2*, *Lachnospiraceae_XPB1014_group*, and *Christensenellaceae_R-7_group*; and those in WB were *Lactobacillus*, *norank_f__Bacteroidales_S24-7_group* and *Clostridium_sensu_stricto_1* (Fig. 8d).

**Fig. 9:**
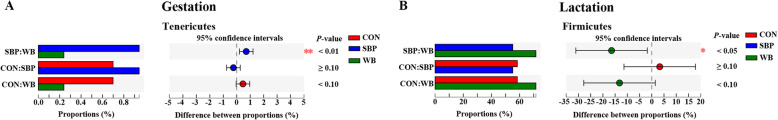
Relative abundance of significantly different phyla on d 110 of gestation (**a**) and d 21 of lactation (**b**). *n* = 5. CON, control diet; SBP, sugar beet pulp diet; WB, wheat bran diet

**Fig. 10:**
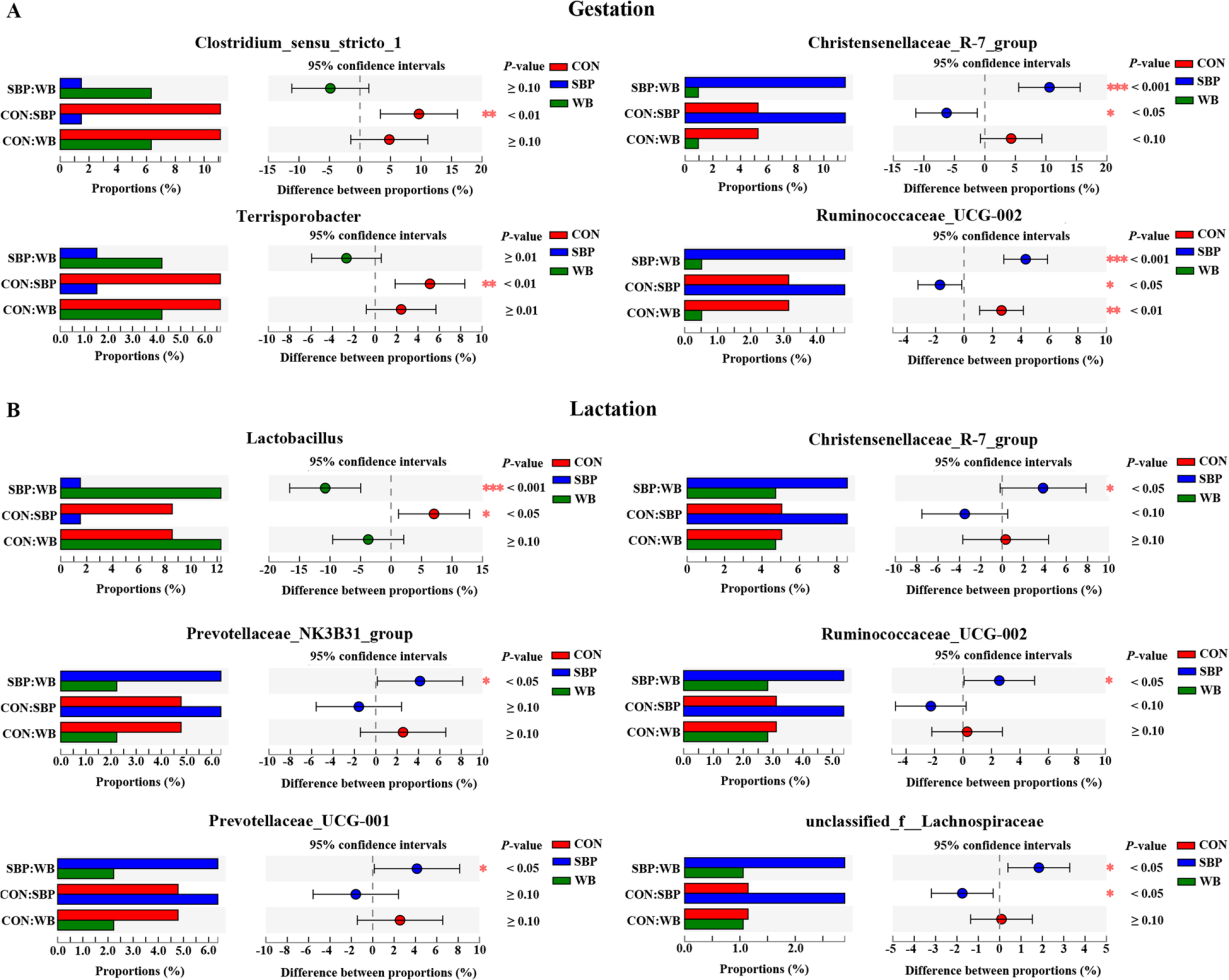
Relative abundance of significantly different genera on d 110 of gestation (**a**) and d 21 of lactation (**b**). *n* = 5. CON, control diet; SBP, sugar beet pulp diet; WB, wheat bran diet

Differential analysis of microbial composition among treatments were further explored. At the phylum level, sows fed SBP had greater (*P* < 0.05) abundance of phyla Treponema than those fed WB on d 110 of gestation (Fig. 9a), while supplementation of WB enriched (*P* < 0.05) the abundance of Firmicutes compared with SBP on d 21 of lactation (Fig. 9b). At the genus level, on d 110 of gestation, the SBP supplementation significantly decreased (*P* < 0.01) the abundance of *Clostridium_sensu_stricto_1* and *Terrisporobacter* compared with CON (Fig. 10a). Sows fed SBP had greater (*P* < 0.05) abundance of *Christensenellaceae_R-7_group* and *Ruminococcaceae_UCG-002* than those fed the other two diets. In addition, supplementation of WB reduced (*P* < 0.01) the abundance of *Ruminococcaceae_UCG-002* when compared with CON. On d 21 of lactation, sows fed WB and CON had greater (*P* < 0.05) abundance of *Lactobacillus* than those fed SBP (Fig. 10b). Compared with WB, the SBP supplementation enriched (*P* < 0.05) the abundance of *Christensenellaceae_R-7_group*, *Prevotellaceae_NK3B31_group*, *Ruminococcaceae_UCG-002*, and *Prevotellaceae_UCG_001*. In addition, sows fed SBP had greater (*P* < 0.05) abundance of *unclassified_f__Lachnospiraceae* than those fed the other two diets.

### Fecal short chain fatty acids

**Fig. 11:**
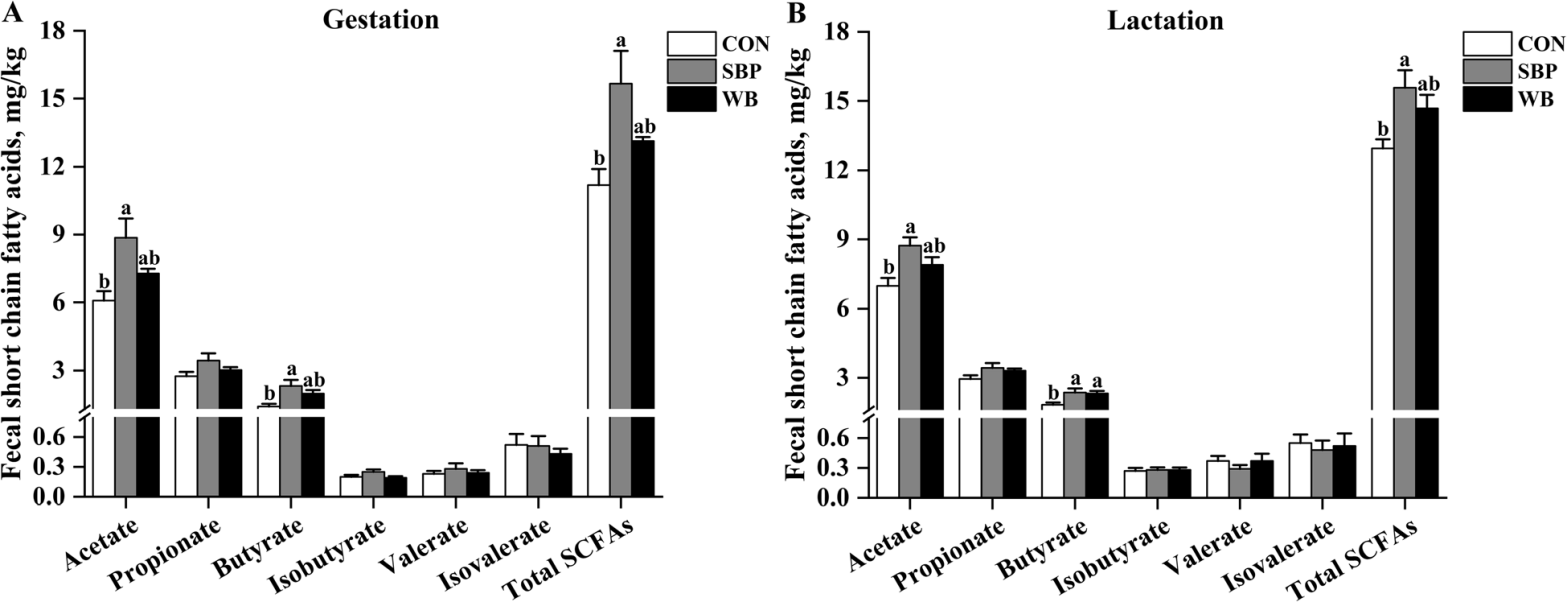
Effects of fiber sources on fecal short chain fatty acids in sows. **a-b** Fecal short chain fatty acids on d 110 of gestation and d 21 of lactation. Date were presented as mean ± SEM, *n* = 5. Different letters mean significant differences (*P* < 0.05). SCFAs, short chain fatty acids; CON, control diet; SBP, sugar beet pulp diet; WB, wheat bran diet

Effects of fiber sources on fecal short chain fatty acids in sows are presented in Fig. 11. On d 110 of gestation, fecal concentrations of acetate, butyrate and total SCFAs were increased (*P* < 0.05) in sows fed SBP diets compared with those fed CON diets, but not different from those fed WB diets (Fig. 11a). There were no differences observed in fecal concentrations of propionate, isobutyrate, valerate and isovalerate among treatments. On d 21 of lactation, compared with sows fed CON diets, sows fed SBP diets had greater (*P* < 0.05) fecal concentrations of acetate, butyrate and total SCFAs, while sows fed WB diets had greater (*P* < 0.05) fecal concentration of butyrate (Fig. 11b). There were also no differences in fecal concentrations of propionate, isobutyrate, valerate and isovalerate among treatments.

## Discussion

During lactation, sows mobilize their body reserves to support milk synthesis, which generally leads to body loss at weaning [21]. Sow body condition at weaning is known to be closely associated with its reproductive performance as a good body condition plays a vital role in maintaining a good reproductive performance, while in contrast, a poor body condition has adverse impacts on the subsequent reproduction performance by prolonging weaning-estrous interval and decreasing litter sizes [22]. In the present study, sows fed SBP showed lower lactation weight loss than those fed CON, suggesting a better body condition. Similarly, Renteria-Flores et al. [23] also observed a lower lactation weight loss in sows fed high fiber diet compared with those fed the control diet. However, the results were not always consistent as some researches failed to detect positive effects of high fiber diets on lactation weight loss in sows [24]. The discrepancies for the consistent results may be due to the sources, inclusion levels of fiber as well as feeding duration and stage of animals [13, 25].

It is well known that constipation is a common symptom for pregnant sows because gastrointestinal motility was decreased, and transit time was significantly prolonged during pregnancy, thereby resulting in increased water absorption and eventually low frequency and hard stools [26]. Constipation can lead to a series of distressing symptoms, including abdominal distension, gut obstruction, perforation, and increased farrowing duration, thereby affecting health of sows [27]. In this study, greater fecal water content was observed in sows fed SBP and WB diets when compared with those fed CON diets, suggesting that high fiber diets may alleviate the constipation severity in pregnant sows by retaining fecal water content. Our results were in consistent with previous studies, in which high fiber diets containing konjac flour or alfalfa meal increased fecal water content and relieved constipation in pregnant sows [28, 29]. Dietary fiber generally has great water-binding capacity, and also can reduce transit time and increase stools bulk, which may contribute to the alleviative constipation [30, 31].

Serum biochemical parameters are useful biomarkers for monitoring body health and physiological condition [32]. Protein that escapes digestion in the foregut is fermented partly in the hindgut into ammonia, which is either used as nitrogen source for microbiota or absorbed into blood and transformed to urea in the liver [33, 34]. Therefore, blood urea nitrogen can reflect nitrogen utilization efficiency in various animal species [35]. In this study, sows fed SBP diets showed lower serum urea nitrogen concentration compared with those fed CON diets during gestation, indicating greater nitrogen utilization efficiency in sows fed SBP. Similarly, previous studies also revealed that fermentable fiber could reduce plasma urea nitrogen in growing pigs and sows [25, 36]. One possible explanation is that as substrates for bacteria, fermentable fiber can increase bacterial mass, which, in turn, utilizes more ammonia as nitrogen for protein synthesis, thereby reducing urea nitrogen absorption into blood [36]. Another possible explanation is that dietary fiber can suppress protein fermentation, thereby reducing ammonia production [37]. However, a part of our results indicated that sows fed WB diets did not show lower serum urea nitrogen concentration compared with CON most likely because soluble fiber has a greater capacity to increase microbial mass and activity in comparison with insoluble fiber [38].

An interesting finding in this study is that sows fed both SBP and WB had lower serum TC concentration compared with those fed CON during gestation. Our results were in consistent with Ndou et al. [39], in which both soluble fiber (flaxseed meal) and insoluble fiber (oat hulls) decreased serum total cholesterol concentration in pigs, showing hypocholesterolemic effects. As a source of soluble fiber, the SBP can increase the water holding capacity of digesta, and hence increase cholesterol and bile acid excretion, which may in turn influence hepatic cholesterol metabolism, and eventually result in decreased serum cholesterol concentration [40]. While as a source of insoluble fiber, the lignin present in WB can increase cholesterol and bile acid excretion in the gastrointestinal tract [39].

It is well known that NEFA are a product of fat metabolism and a good indicator of catabolism of fat reserves [41]. In the current study, the decreased serum NEFA concentration observed in sows fed SBP might suggest reduced fat metabolism, and therefore better body reserve. Indeed, this study demonstrated that sows fed SBP had a lower body loss during lactation though no significant difference was observed in backfat loss among treatments. In contrast, the WB supplementation did not influence serum NEFA concentration when compared with CON. It has been shown that the SCFAs production was negatively correlation with serum NEFA concentration and fermentable fiber could decrease serum NEFA concentration by increasing SCFAs production [42]. The SBP contains more soluble fibers (e.g. pectin) that are readily fermentable than WB, therefore, more SCFAs were produced in sows fed SBP as indicated by increased fecal concentration of total SCFAs.

Pregnancy is generally associated with a systemic inflammatory response, which has adverse effects on both maternal and fetal health [1]. In the current study, lower serum concentration of pro-inflammatory cytokine IL-6 was observed in sows fed SBP and WB during pregnancy and lactation, suggesting alleviative inflammatory responses by fiber supplementation. In addition, sows fed SBP also showed lower serum concentration of pro-inflammatory cytokine TNF-α and greater serum concentration of anti-inflammatory cytokine IL-10 than those fed CON, indicating that SBP may be more effective in reducing inflammation than WB. It is known that IL-10, an anti-inflammatory cytokine, can suppress proinflammatory responses by decreasing cytokine and chemokine production [43]. Thereby, the SBP may relieve inflammation by increasing the production of anti-inflammatory cytokine IL-10. Likewise, previous studies also found that higher intake of dietary fiber was closely related to decreased severity of inflammation, in contrast, dietary fiber deprivation resulted in inflammation and increased pathogen susceptibility [44–46]. The positive effects of dietary fiber on inflammation observed in this study may be correlated to the changed gut microbiota and their by-products induced by the two sources of fiber supplementation [47].

Intestinal microbiota plays a crucial role in maintaining host health by regulating metabolism and immune system [48]. In the present study, even if there were no significant changes in α-diversity among three treatments within each period, principal coordinates analysis (PCoA) showed a distinct clustering for each dietary treatment at both periods, illustrating microbial composition changed differently in response to different fiber sources. Generally, Firmicutes and Bacteroidetes were the most dominant phyla in most mammals [49]. In this study, Firmicutes and Bacteroidetes were indeed the most abundant phyla in all treatments regardless of the periods, which was consistent with previous studies [50, 51]. We further found that sows fed SBP showed an increased abundance in phyla Tenericutes compared with WB during gestation. Tenericutes has been reported to have the ability to improve lipid metabolism and blood lipid profiles [52, 53]. Therefore, the increased abundance of Tenericutes may be associated with the decreased serum concentrations of TC and NEFA in sows fed SBP. In addition, gut inflammation has been shown to result in a significant reduction in the abundance of Tenericutes [54, 55]. Thus, the high abundance of Tenericutes in SBP-fed sows may be positively correlated with the decreased serum concentrations of pro-inflammatory cytokines (IL-6 and TNF-α). Interestingly, supplementation of WB increased the abundance of Firmicutes compared with SBP during lactation. It has been shown that an increased abundance in Firmicutes is generally associated with a greater capacity of energy absorption from the diet and a greater feed conversion ratio [56, 57]. The increased abundance of Firmicutes during lactation would be thus desired because lactating sows need more energy to maintain the requirements of themselves and their offspring. But unexpectedly sows in SBP rather than those in WB had lower lactation BW loss compared with CON, indicating gut microbiota was not the only factor involved in regulating lactation BW loss. Lactation feed intake has been reported to be a critical factor to influence sow body condition, and increasing lactation feed intake could minimize weight loss [58]. Soluble dietary fiber has been shown to be more effective than insoluble fiber in improving lactation feed intake, which may explain the lower lactation BW loss in sows fed SBP [59].

Certain changes were also observed at the genus level in sows fed different fiber sources. During gestation, the SBP supplementation significantly decreased the abundance of *Clostridium_sensu_stricto_1* compared with CON. *Clostridium_sensu_stricto_1* is generally considered as pathogenic bacteria and growing evidence indicates that it is closely associated with inflammation [60]. It has been shown that the mRNA expression of TNF-α and IL-1β was positively correlated with the enrichment of *Clostridium_sensu_stricto_1*, and this enrichment eventually resulted in colonic inflammation [61]. Moreover, the increased abundance of *Clostridium_sensu_stricto_1* was also observed in other inflammatory models (e.g. endometritis, necrotizing enterocolitis or lipopolysaccharide-induced gut inflammation) [62–64]. As a result, the decreased abundance of *Clostridium_sensu_stricto_1* may partly explain the lower serum concentrations of pro-inflammatory cytokines (IL-6 and TNF-α) in sows fed SBP. Our result also showed that sows fed SBP had lower abundance of *Terrisporobacter* and greater abundance of *Christensenellaceae_R-7_group* compared with CON. It has been reported that *Terrisporobacter* is an obesity-promoting bacteria and positively correlated with serum lipids [65]. In contrast, Christensenellaceae has been shown to be negatively correlated with serum lipids [66]. Therefore, the decreased abundance of *Terrisporobacter* and the increased abundance of *Christensenellaceae_R-7_group* may contribute to the improved serum lipid profile in sows fed SBP. Furthermore, the present study demonstrated that sows fed SBP showed greater abundance of *Ruminococcaceae_UCG-002* than those fed the other two diets. Ruminococcaceae is known to produce short-chain fatty acids by degrading various polysaccharides and has shown to be negatively related to inflammation [67]. As a consequence, the increased abundance of *Ruminococcaceae_UCG-002* would be favourable for the reduced inflammatory responses in sows fed SBP.

During lactation, the increased abundance of *Christensenellaceae_R-7_group* and *Ruminococcaceae_UCG-002* was observed in sows fed SBP as well, which may be a contributing factor to the improved serum lipid profile and the reduced serum pro-inflammatory cytokines. In addition, the SBP supplementation also enriched the abundance of *Prevotellaceae_NK3B31_group* and *Prevotellaceae_UCG_001*. *Prevotellaceae_NK3B31_group* has been reported to be negatively correlated with serum concentrations of IL-6 and TNF-α, exhibiting anti-inflammatory properties [68]. Wang et al. [69] also reported that the increased abundance of *Prevotellaceae_NK3B31_group* contributed to alleviate gut inflammation in weaned pigs challenged with enterotoxigenic *Escherichia coli* K88. In addition, *Prevotellaceae_UCG_001* also exerted anti-inflammatory function which has been shown to be positively associated with anti-inflammatory cytokines (e.g. IL-4 and IL-10) and inversely correlated with pro-inflammatory cytokines (e.g. IL-6 and TNF-α) [70]. Therefore, these alterations in microbial composition induced by SBP supplementation may contribute to alleviate inflammatory responses by reducing serum concentrations of IL-6 and TNF-α.

An important finding in this study was that sows fed WB had greater abundance of *Lactobacillus* than those fed SBP during lactation. Moreover, during gestation, sows fed WB also showed high abundance of *Lactobacillus* although no significant difference was observed among treatments. *Lactobacillus* species are well-known probiotics on account of their multiple health-promoting effects, including suppression of intestinal inflammation, enhancement of intestinal barrier function, modulation of immune responses, maintenance of microbial homeostasis and prevention of diseases [71–73]. Therefore, the increased abundance of *Lactobacillus* in sows fed WB may be responsible for the decreased serum IL-6 concentration. These results, taken together, indicated that supplementation of SBP and WB could improve sow health by differently altering microbial composition.

There is growing evidence that microbial metabolite short-chain fatty acids are key executors of diet-based microbial effect on the host [10]. Changes in intestinal microbial composition are generally accompanied by changes in the production of SCFAs [74]. The current study showed that fecal concentrations of acetate, butyrate and SCFAs were increased by SBP supplementation compared with CON during both gestation and lactation, suggesting greater microbial fermentation in the gut. Ruminococcaceae and Lachnospiraceae are well-known butyrate-producing bacteria [75]. *Ruminococcaceae_UCG-002* is reported to produce butyrate by fermenting indigestible carbohydrates, while Christensenellaceae has the ability to produce acetate and butyrate [66, 76]. *Prevotellaceae_NK3B31_group* has been shown to be positively correlated with acetic acid production [77]. Consequently, the increased concentrations of acetate and butyrate in sows fed SBP may be attributed to the increased abundance of *Ruminococcaceae_UCG-002*, *Christensenellaceae_R-7_group*, *Prevotellaceae_NK3B31_group* and *unclassified_f__Lachnospiraceae*. The present study also showed that supplementation of WB increased fecal concentration of butyrate during lactation. It has been shown that lactic acid is used by some butyrate-producing bacteria for the production of butyrate [78], thus the increased abundance of *Lactobacillus* may be, at least in part, indirectly responsible for the significant increase of butyrate. The present study indicated that supplementation of both fiber sources significantly increased fecal concentration of butyrate. Butyrate is the most effective SCFA, which not only provides energy for colonocytes, but also maintain gut homeostasis by inhibiting inflammation and carcinogenesis, reinforcing barrier function and alleviating oxidative stress [79]. As a result, the increased concentration of butyrate may alleviate inflammation and maintain gut health in sows fed either SBP or WB.

## Conclusions

In conclusion, both SBP and WB supplementation could improve metabolism, immune responses and gut health in sows but by differently affecting microbiota. In addition, the SBP was more effective than WB in terms of these indexes.

## Data Availability

All data generated or analyzed during this study are included in this published article.

## Abbreviations

BW: Body weight
CON: Control diet
DF: Dietary fiber
GLU: Glucose
IgA: Immunoglobulins A
IgG: Immunoglobulins G
IgM: Immunoglobulins M
IL-6: Interleukin-6
IL-10: Interleukin-10
NEFA: Non-esterified fatty acids
OUT: Operational taxonomic units
PCoA: Principal coordinate analysis
SBP: Sugar beet pulp diet
SCFAs: Short chain fatty acids
TC: Total cholesterol
TG: Triglyceride
TNF-α: Tumor necrosis factor-α
UN: Urea nitrogen
WB: Wheat bran diet
